# EPR Spectroscopy Provides New Insights into Complex Biological Reaction Mechanisms

**DOI:** 10.1021/acs.jpcb.2c05235

**Published:** 2022-09-22

**Authors:** Lukas Hofmann, Sharon Ruthstein

**Affiliations:** Department of Chemistry and the Institute of Nanotechnology & Advanced Materials, Faculty of Exact Sciences, Bar-Ilan University, Ramat-Gan 5290002, Israel

## Abstract

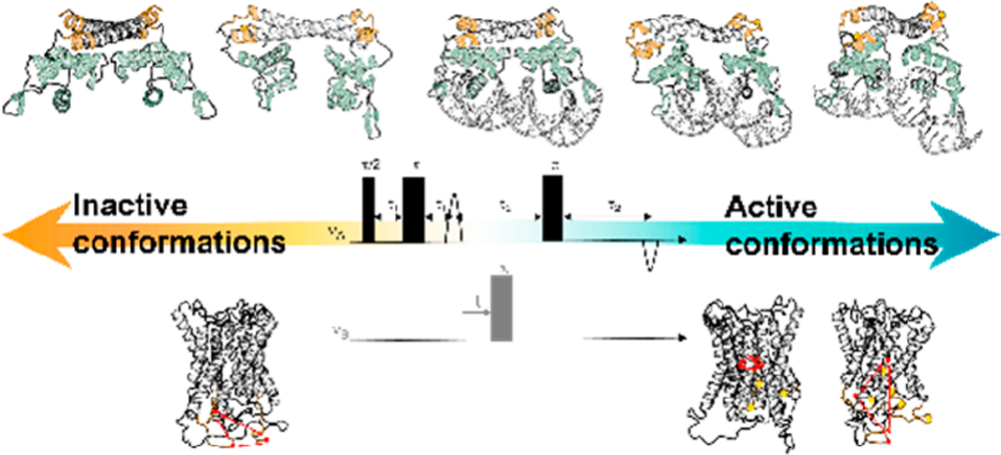

In the last 20 years, the use of electron paramagnetic resonance (EPR) has made a pronounced and lasting impact in the field of structural biology. The advantage of EPR spectroscopy over other structural techniques is its ability to target even minor conformational changes in any biomolecule or macromolecular complex, independent of its size or complexity, or whether it is in solution or in the cell during a biological or chemical reaction. Here, we focus on the use of EPR spectroscopy to study transmembrane transport and transcription mechanisms. We discuss experimental and analytical concerns when referring to studies of two biological reaction mechanisms, namely, transfer of copper ions by the human copper transporter hCtr1 and the mechanism of action of the *Escherichia coli* copper-dependent transcription factor CueR. Last, we elaborate on future avenues in the field of EPR structural biology.

## Introduction

Cellular regulation and cell survival rely, in part, on interactions between soluble and/or membrane proteins and between proteins and other cellular components. Understanding biological reactions that transpire within the cell at the molecular level is essential for developing novel therapeutic approaches. Structural biology plays a dominant role in understanding structure–function relationships of proteins in the cell. The most common structural biology tools in use today are X-ray crystallography, nuclear magnetic resonance (NMR) spectroscopy, and electron microscopy. While each of these methods has its own pros and cons, they all struggle with how to gain information on complex biological systems, such as how transcription factors elicit gene expression and how transporters deliver ligands and small molecules across membranes. Here, we will focus on these two structurally challenging biological systems and show the benefits of using electron paramagnetic resonance (EPR) spectroscopy as a biophysical tool for resolving reaction mechanisms in complex biological systems.

DNA-binding proteins are essential for many aspects of genetic activity, such as homeostasis, transcription regulation, DNA conformation, replication, and cell repair. It is, therefore, essential to examine the nature of how complexes are formed between proteins and DNA, such as RNA polymerases (RNAp), since the steps of complex assembly form the basis of our understanding of how these processes are regulated. Over recent decades, we have witnessed a great expansion in the number of resolved high-quality structures of DNA- and RNA-binding proteins and their nucleic acid targets. The first protein–DNA structure to be solved was that involving the *Escherichia coli* catabolite activator protein in 1981. Four decades later, the Protein Data Bank contains some 1000 structures of protein–DNA complexes. Most of these structures have been obtained using X-ray crystallography and NMR. The structures of such complexes have provided valuable insight into the principles of protein–DNA binding, including how specific DNA bases are recognized and how DNA structures are modified upon protein binding. More recently, the field of X-ray protein crystallography has been complemented by electron microscopy, allowing us to resolve the structures of protein–DNA–RNAp complexes, membrane proteins, and large complexes.^1^ Of these, the transmembrane proteins are of particular interest because they provide the cell with gates to its surroundings. Accordingly, transmembrane proteins can allow the cell to appropriately respond to the environment, as in the case of G protein-coupled receptors (GPCRs), or act as import or export transporters which establish an essential flow of nutrients, salts, energy and more.^2,3^ In addition to providing functions essential for cell survival, these groups of proteins also represent a point of entry into the cell and thus represent important targets for drug discovery.

To date, more than 7300 membrane protein structures have been reported, of which about 500 consist of β-sheets and 6700 of α-helical structures, according to the Research Collaboratory for Structural Bioinformatics Protein Data Bank (RCSB PDB). Yet, these membrane proteins only represent a minor fraction of the 200 000 structures reported in RCSB PDB. This disparity is indicative of the fact that gaining structural insight into membrane proteins and complexes thereof remains a challenge. Indeed, major efforts will be required to ultimately reveal the secrets of the mechanisms of action of such entities. It is clear that by deciphering the form or structural changes of a membrane protein, the underlying function can be deduced and subsequently targeted as part of drug discovery attempts. In what follows, we briefly discuss four methodologies that can help in such efforts, with an emphasis on EPR spectroscopy, as applied to deciphering the mechanisms used by transporters and transcription factors.

NMR spectroscopy represents a powerful approach to answer current questions on intricate biological mechanisms. Liquid-state NMR experiments are capable of detecting interactions between proteins and small molecules, as well as following metabolic processes, riboswitches, and even protein phosphorylation.^4,5^ However, the use of liquid NMR is limited by the size of the biological system of interest. Specifically, it is currently highly challenging to employ this approach for the study of large and complex biological systems, such as membrane transporters and transcription processes, which involves DNA, RNA, ligands, small molecules or ions, and proteins. While solid-state NMR can provide useful information on large membrane proteins,^6^ deducing information on the dynamics of transfer via a transporter remains challenging. Förster or fluorescence resonance energy transfer (FRET) can overcome some of the limitations that restrict NMR. The power of FRET lies in its ability to report intermolecular interactions at the nanometer scale (1–10 nm). In general, FRET measurements are sufficiently accurate to describe kinetic parameters, overall mechanistic transitions,^7^ and time scales, yet they cannot explain the fundamental mechanical driving forces nor provide accurate topological changes of structural rearrangements, data which are essential for following complex biological reaction mechanisms.^7^ Cryo-electron microscopy (cryo-EM) and cryo-electron tomography (cryo-ET) are becoming major tools for determining protein structures at high-resolution.^8^ Currently, the main advantage of cryo-EM/ET is that structures of large complexes or membrane proteins collected by these approaches can be resolved. However, it should be kept in mind that those systems investigated thus far contain highly abundant proteins or complexes with high symmetry. At present, cryo-EM/ET is challenged for monitoring proteins of low abundance and low symmetry *in vivo*. Moreover, the use of these technique encounters difficulties in differentiating between close conformational states or changes in the dynamics of protein domains.

In the past decade, EPR spectroscopy has emerged as an excellent methodology for following biological mechanisms. The use of EPR spectroscopy does not require crystallization and is not limited by protein size. Moreover, EPR spectroscopy allows for detecting proteins in solution without the need to isotope-label the biomolecule, as is the case with NMR spectroscopy. The advantages of EPR spectroscopy, as compared to other methods, include higher sensitivity that allows for the monitoring of minor conformational changes in a targeted biomolecule and the fact that it is unlimited in terms of the size and/or complexity of a biological system or its environment. Additionally, EPR can target a biomolecule found at concentrations as low as the micromolar range.^9^ The basic principle of EPR spectroscopy is the measurement of unpaired electron spins of a given molecule. Since most proteins lack these intrinsic radicals, it is possible to tag them with a paramagnetic probe, known as a spin-label. There are several well-established spin-labeling approaches both for proteins and for DNA/RNA, as discussed below.

## Methods

### Protein Spin-Labeling Approaches

**Figure 1:**
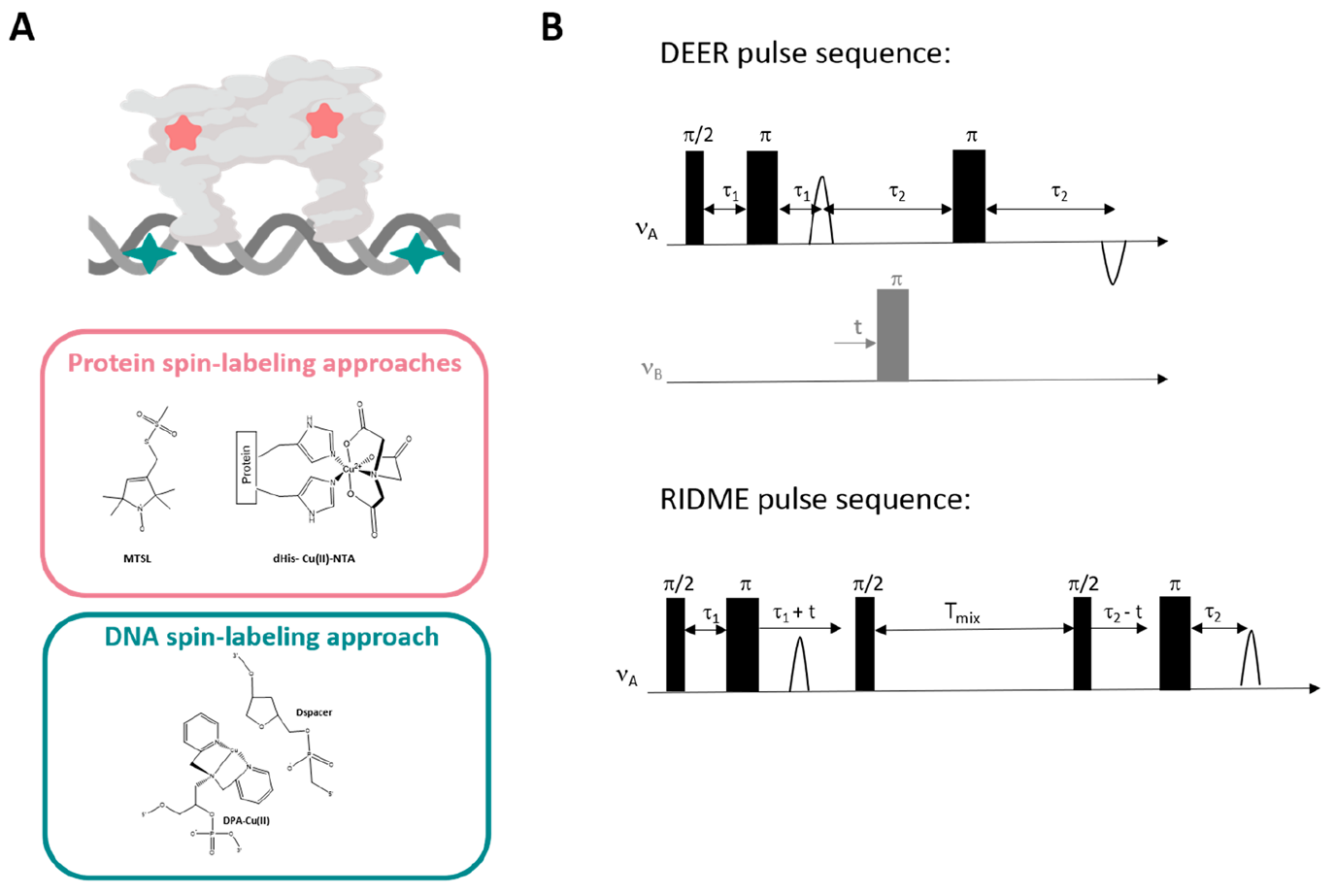
(A) Examples of spin-labeling approaches used in the study of proteins described here (MTSL and Cu(II)–NTA–dHis), and for DNA spin-labeling (Cu(II)–DPA). (B) Examples of pulse sequences of EPR distance measurements.

The most often-used spin-labels are nitroxide radicals, with an electron spin of ^1^/_2_ and a nuclear spin of 1 (corresponds to ^14^N).^10,11^ The most widely used nitroxide spin-label is (1-oxyl-2,2,5,5-tetramethylpyrroline-3-methyl)methanethiosulfonate (MTSL) (Figure 1A), which can be chemically attached to the thiol group of a cysteine residue.^12−14^ This strategy usually requires generation of a mutant lacking all native cysteine residues together with the introduction of at least one cysteine residue via site-directed mutagenesis. The cysteine thiol groups specifically react with functional groups of the spin-label that create a covalent bond with the amino acid. Another nitroxide spin-label that can be attached to cysteine residues is 3-maleimido-2,2,5,5-tetramethyl-1-pyrrolidinylox (MSL), which contains a maleimide group and is slightly more stable than MTSL in a reducing environment. Nitroxide spin-labels can also be attached to sugars, nitrogenous bases, or phosphate backbones via linkers and can, therefore, report on conformational changes in DNA and RNA molecules.^15^ However, these spin-labeling approaches demand sophisticated organic synthesis skills and equipment. Hence, efforts are being devoted to developing novel spin-labeling techniques that require less elaborate postsynthesis modifications.

Other non-nitroxide spin-labels that can be attached to cysteine residues are triarylmethyl (trityl) radicals, which are highly stable even in reducing cellular environments.^16−18^ These are much larger spin-labels, which limit the choice of labeling sites, unlike nitroxide spin-labels.

Paramagnetic metal ions have also been developed as spin-labels for structural measurements. Spin-labels based on Cu(II), Gd(III), and Mn(II) have appeared as alternatives to traditional nitroxides, and have been proven to have advantageous properties in certain cases.^19−21^ Saxena and colleagues recently developed an alternative methodology for adding a Cu(II) center to the backbone of a protein (Figure 1A).^22,23^ As part of their approach, a double histidine (dHis) mutation is introduced to site-specifically attach the Cu(II) ion to the protein. This method shows optimal performance when the dHis site is placed between the *i* and *i* + 4 amino acids in an α-helix and when Cu(II) is bound to a nitriloacetic acid (NTA) ligand, thus avoiding nonspecific binding. In this case, the position of Cu(II) is significantly restricted by coordination to the protein side chain. The resulting distances are, therefore, remarkably precise, with a distance distribution width that is five times narrower than that of a nitroxide spin-label.

In addition, the Saxena group has recently developed another methodology that relies on Cu(II) ions for DNA labeling.^23,24^ In this method, commercially available 2,2′-dipicolylamine (DPA) phosphormadite is easily incorporated into any DNA oligonucleotide during initial DNA synthesis (Figure 1A). The opposing strand uses a dSpacer, which is a commercially available sugar–phosphate backbone devoid of the nucleobase. Hence, this dSpacer can accommodate the bulkier DPA, allowing the formation of spin-labeled double-stranded DNA for EPR measurements.

### Distance EPR Measurements

Double electron–electron resonance (DEER), also known as PELDOR, is the most widely used technique for EPR distance measurement (Figure 1B). A DEER experiment uses two microwave frequencies, one to pump coupled spins and the other to observe any effect on the refocused echo. The echo is modulated by the dipolar coupling frequency, which can then be analyzed using distance distribution functions. A recent manuscript provided detailed guidelines on how to conduct and analyze a DEER experiment.^9^ We will briefly summarize some of the key criteria that need to be considered:

#### Sensitivity

Most DEER experiments involving proteins are performed at X-band (∼9 GHz) frequencies, although the signal-to-nose ratio (SNR) improves at Q-band frequencies (∼34 GHz), specifically when using a high-power setup and a probe head that allows oversized samples. Moreover, the use of arbitrary waveform generator (AWG) can increase the sensitivity by using different pulse shapes.

#### Resolution

To attain a precise and narrow distance distribution, it is important to reduce the contribution of the homogeneous background to the DEER trace as much as possible, which can be achieved by working with a low protein concentration. In our experiments, we have observed that for MTSL spin-labels, it is possible to use labeled-protein concentrations as low as 5–10 μM. For Cu(II)-based spin-labels, it is necessary to use about 50 μM labeled protein. Recently, the use of submicromolar concentrations with Cu(II)–NTA spin-labels was reported.^25^

#### Temperature

DEER experiments using nitroxide spin-labels can be performed either at 80 K using liquid nitrogen or at 50 K using liquid helium. Although using liquid helium is much more expensive, the gain in SNR is significant and can reach up to 4-fold. DEER experiments on Cu(II) spin-labels are best performed at 20 K owing to the fast relaxation time.

#### Functional Tests

When exploring protein–DNA interactions using EPR spectroscopy, it is important to initially verify that the spin-labeled protein is fully functional, or, when labeling the DNA, that the protein can bind the spin-labeled DNA in a similar manner as to non-spin-labeled DNA. To this end, the researcher should first carry out various biochemical experiments, such as runoff transcription and pull-down assays and/or an electrophoretic mobility shift assay (EMSA) for protein–DNA systems. Moreover, circular dichroism measurements should be conducted to verify that the secondary structure of the spin-labeled protein was not affected by spin-labeling.

#### Data Analysis

Nowadays, there are several analysis programs available for assessing DEER data written in Python and in MATLAB. Examples are DeerLab,^26^ DeerNet,^27,28^ and DeerAnalysis.^29^ The DEER time domain can be converted into distance distributions using a variety of models such as Gaussian model distribution, Tikhonov regularization, and others. All of these require that the contribution of the background signal be first subtracted. It is, however, recommended that a single-step method that accounts for both the distance distribution and the background signal be used.^29^

#### Orientation Effects

Data analysis addressing distance distributions (such as achieved using the DeerAnalysis program) neglects orientation effects of the paramagnetic center with respect to the magnetic field, which is good for nitroxide spin-labels. However, this feature might confound measurements for paramagnetic metal ions or rigid spin-labels, in which orientation selection can occur. It is possible to limit such bias by obtaining all DEER traces in a fixed magnetic field. For Cu(II) spin-labels, fixing the magnetic field at g_perp_ will result in minimal orientation dependence.

#### Other Pulsed EPR Distance Measurement Experiments

DEER experiments are highly suitable for measuring distances between two nitroxide radicals; however, when the EPR spectrum is much broader, such as with the Cu(II) ion spectrum, then a DEER experiment is limited by the excitation bandwidth, owing to the use of two microwave frequencies. This can be overcome by relaxation-induced dipolar modulation enhancement (RIDME). RIDME (Figure 1B) relies on the coupled center undergoing longitudinal relaxation to modulate the signal of the coupled spin centers.^30,31^ The background contribution in a RIDME experiment is higher, which, therefore, affects the modulated echo signal. At the same time, artifacts arising from improper phase cycles can also affect the acquired results. RIDME experiments hold a distinct advantage over DEER experiments, especially for Cu(II)–nitroxide measurements in a Q-band measurement, owing to the limitation of bandwidth excitation in the Q-band, the greater sensitivity, and the longer time domain signal attained by RIDME than by DEER.

### Structure Modeling

One of the limitations of EPR spectroscopy is the inability to determine the three-dimensional structure of a protein based on collected distance distribution constraints, such that no PDB file can be deposited. However, if a PDB structure of the studied protein or its homologue is available, this can serve as the basis for structural modeling using EPR distance constraints. Alternatively, alphafold2 structures can also be used as a preliminary PDB file.^32^ In this case, the calculated models are structures or conformations that the protein assumes during a biological mechanism in solution, thus providing detailed insight into the mode of action. In recent years, several programs have been developed for the EPR community, such as the rotamer library approach (named elastic network model, ENM) implemented in the MMM program,^33^ mtsslWizard,^34^ or ALLNOX.^35^ All are simple to use and can derive structural models based on the various distance distribution constraints obtained by EPR measurements. Moreover, molecular dynamics (MD) simulations can also be applied to model dynamic processes with restraints derived from experimentally derived EPR measurements.^14^

## Results and Discussion

In the past decade, there has also been a breakthrough in the use of EPR spectroscopy to study complex biological systems that had not previously resolved by other conventional tools. In this manner, considerable progress was in studying the gating mechanisms of membrane transporters, such as the ABC transporters.^36^ A combination of DEER experiments using a variety of spin-labeling approaches with cryo-EM and MD simulations shed light on intermediate structures realized during ATP binding which could not have been obtained by other methods.^37^ EPR experiments were used to target conformational and dynamical changes of the MscL ion channel.^38^ Moreover, following *in situ* conformational changes of membrane transporters in a lipid environment^39^ and in intact cells^40^ opened many new routes for understanding mechanisms of ligand and ion transfer across the membrane. Electron spin–echo envelope modulation (ESEEM) spectroscopy has also been used to provide information on the gating mechanism of transporters and channels. ESEEM can evaluate the interaction between the electron spin and nearby nuclei,^41,42^ and therefore, it can be used to measure solvent accessibility using deuterated solvent in large membrane proteins. For instance, the combination of DEER and ESEEM experiments jointly provided a vital information on the activation of MscL channel.^43,44^ Thus, EPR measurements themselves and the ability to combine these different EPR methodologies allow to provide a comprehensive understanding of vital molecular mechanisms found in countless biological systems.

EPR measurements were also used to monitor protein–DNA interactions.^45−47^ Qin and co-workers used nitroxide spin-labeled DNA to understand the mechanism of action of the CRISPR-associated Cas9 protein, and they successfully targeted changes in DNA flexibility that occurred during the cleavage process.^48^

The advantages of EPR spectroscopy to study transfer mechanisms and transcription regulation have also been exploited in our lab. We now describe two examples emphasizing the power and complementary insight provided by EPR spectroscopy in biophysical research.

### The Mechanism of Copper Transfer through the Human hCtr1 Transporter

**Figure 2:**
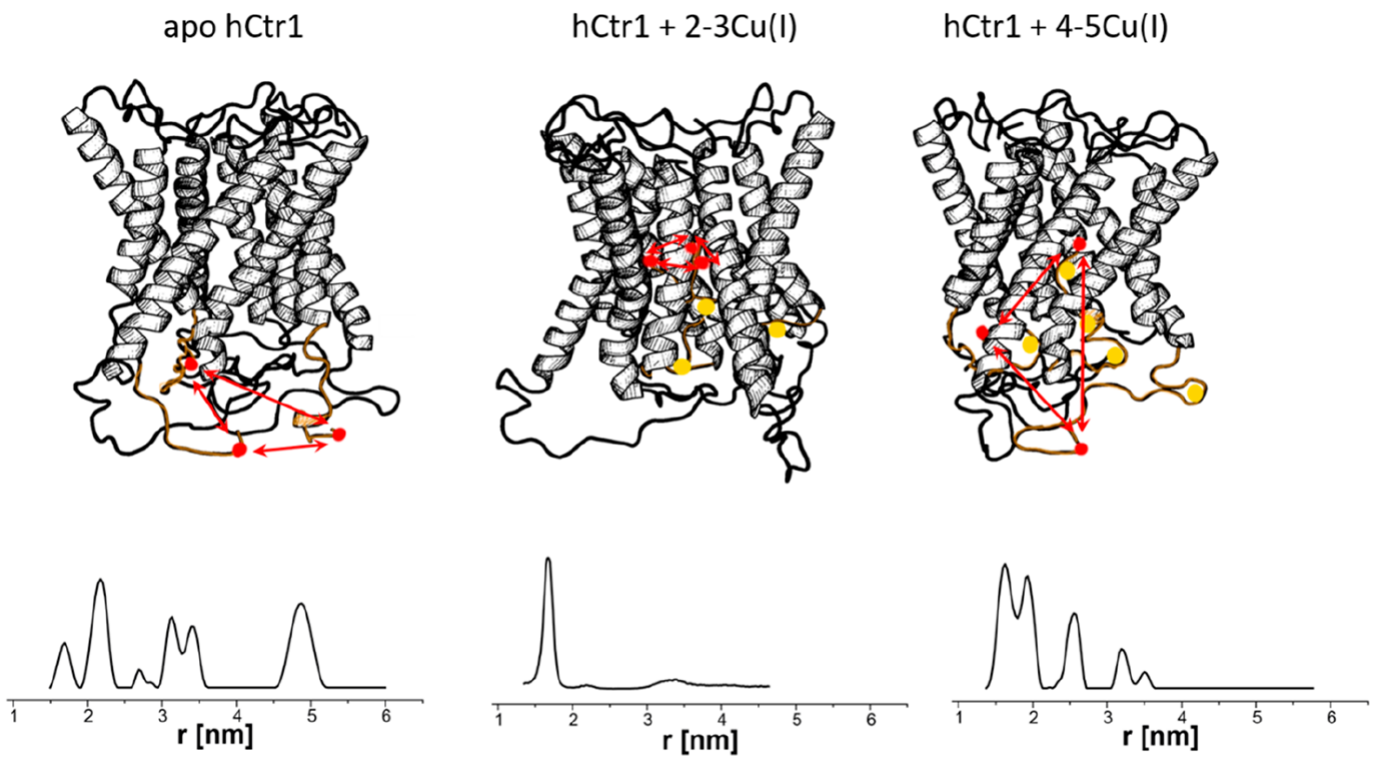
Changes in the EPR distance distribution functions of hCtr1 at various copper concentrations (adapted with permission from ref (14), copyright 2022 Cell Press). Copper-binding sites at the C-termini are colored in orange, copper atoms are indicated as yellow dots. The red arrows show the distances between the C-termini at different copper concentrations.

Copper is required for many important chemical and biological reactions in the cell. However, owing to its ability to undergo oxidation–reduction exactions, it can lead to toxicity and cell death. Therefore, cells have evolved sophisticated regulation mechanisms to control intracellular copper concentrations. The main copper transporter in the human cell is hCtr1. hCtr1 serves various roles, such as acquiring copper in the Cu(II) oxidation state from blood carrier proteins^14,49^ and reducing Cu(II) to Cu(I) and transferring it to various pathways in the cells (such ATP7A/B in the Golgi apparatus, or superoxide dismutase (SOD) and cytochrome c in mitochondria). The extracellular domain of hCtr1 contains histidine and methionine residues that coordinate Cu(II) and Cu(I) ions. As hCtr1 expression and purification are challenging, only limited structural information on this protein is available. The crystal and cryo-EM structures suggest hCtr1 to be a trimer, with each monomer containing three transmembrane helices. The extracellular and intercellular domains of hCtr1 have been less studied owing to their disordered segments. These domains play critical functions in the mechanism of copper transport. We succeeded in expressing and purifying the complete hCtr1 protein from insect cells. Using EPR and UV–vis measurements, we demonstrated that each hCtr1 monomer can coordinate two Cu(II) ions and up to five Cu(I) ions, proposed as reflecting the continuous transfer of copper ions into the cell. To obtain information on the transfer mechanism, the C-terminus of hCtr1, which resides within the cytosol, was spin-labeled with MTSL. Changes in the C-terminal domain were then monitored in DEER experiments. The data presented in Figure 2 suggested various distance distribution functions between 1.5 and 6.0 nm for spin-labeled native hCtr1. The addition of Cu(I) affected these distributions. Interestingly, a single distance distribution at 1.6 ± 0.3 nm was obtained at a ratio of 3Cu(I):hCtr1 monomer. This suggested that, at this copper concentration, all three C-terminal domains were localized in a homogeneous and symmetric manner, with respect to each other (Figure 2). To further our understanding, we ran quantum mechanics/molecular mechanics (QM/MM) simulations. These simulations suggested that the C-terminus interacts with the hCtr1 transmembrane pore domain, allowing for improved copper transfer into the cells. When the copper concentration was further increased, some of the C-termini were released, and an increase in the distance distribution functions was observed.

Taken together, these efforts allowed us to gain functional information on the copper transfer mechanism through the hCtr1 transporter through EPR and QM/MM simulations. To better our understanding of the transport mechanism involved, additional labeling of the extracellular and transmembrane domains of hCtr1 should be carried out, as is in progress in our lab.

### Metal Transcription Factors

Metal transcription factors are proteins that regulate intracellular metal concentrations in bacteria.^13,24,50,51^ These proteins have evolved metal coordination sites that recognize and complex specific metals ions. This binding, in turn, activates or inhibits DNA binding or transcription activation, ultimately controlling the expression of genes that mediate exquisitely selective adaptive responses to elevated metal concentrations. The metal selectivity of metal transcription factors is defined by the coordination chemistry of the chelate, combined with the ability of the chelate to induce changes in protein structures and/or dynamics to affect biological regulation. Understanding these exact mechanisms of action are crucial to elucidate how bacteria maintain metal homeostasis and to develop novel antibiotics based on metal dysregulation. In this perspective, we will focus on a specific metal transcription factor, *E. coli* CueR, and will demonstrate how EPR spectroscopy can provide comprehensive understanding of its mechanism of action.

CueR protein is a member of the MerR family of metal-sensing transcriptional regulators.^52−54^ MerR family proteins exist in most bacterial species and share similar structures and sequences. Hence, understanding the structure–function relationship of a representative protein will provide insight into the functioning of the entire MerR family.

**Figure 3:**
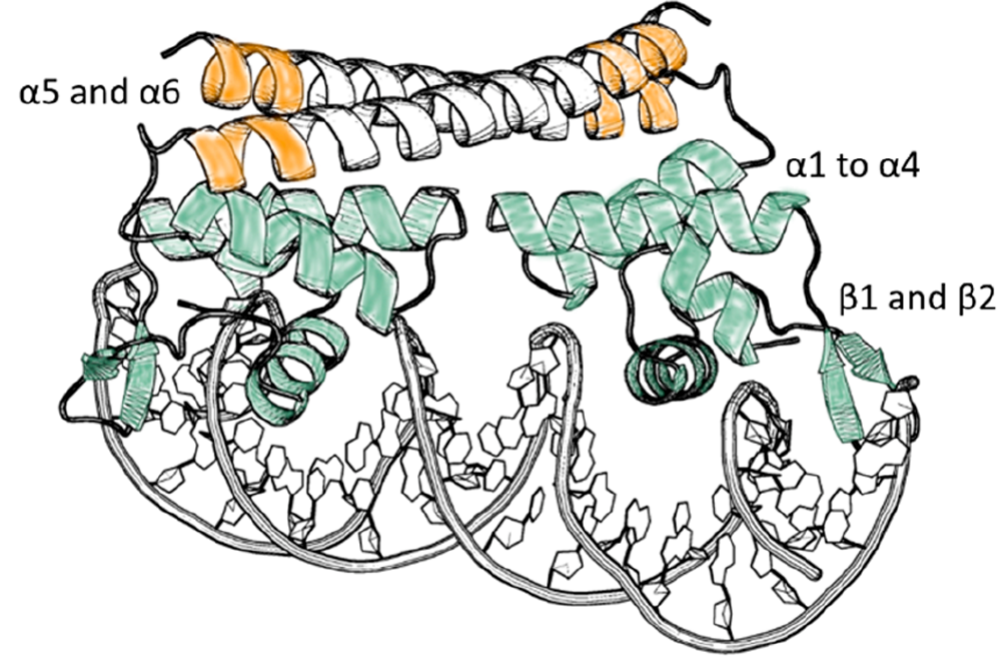
CueR structure (PDB 4WLS) in the repressed state. The green region marks the DNA-binding domain, while the yellow marks represent the C112 and C120 residues involved in Cu(I) binding.

Figure 3 shows the crystal structure of the CueR protein in association with DNA. This interaction involves Cu(I)-binding sites between the α5 and α6 helices, and a DNA-binding domain comprising the β1−β2 and α1−α4 helices.^54^ Copper binding by the CueR-DNA complex induces transcription of two proteins involved in copper homeostasis in *E. coli*. Interestingly, in the absence of metal ions, the metal-dependent regulator CueR prevents constructive interference with RNAp by bending the DNA promoter region in an unfavorable conformation and thus repressing transcription.^53,55^ Upon metal coordination, the DNA is believed to assume a second, different conformation whereby RNAp can successfully interact with the DNA to initiate the transcription process.

Using single-molecule fluorescent resonance energy transfer (smFRET), the Chen group showed that, in solution, CueR can exist in four different states, namely, apo-CueR, holo-CueR, apo-CueR bound to DNA, and holo-CueR bound to DNA. Activation and repression of the transcription process occur when DNA was bound to holo-CueR and apo-CueR, respectively.^56^ However, these smFRET experiments were performed on only single labeling positions in the DNA and CueR, and thus they could not offer a clear model on the structural changes that underlie transcription initiation.^57,58^ The crystal structure of CueR demonstrated that the major difference between the apo- and holo-states is found in the DNA conformation. These DNA conformations are stabilized by two slightly different conformations of CueR. Therefore, it was not possible to obtain comprehensive understanding of the transcription initiation mechanism controlled through CueR by X-ray crystallography.

### Following Conformational Changes of *E. coli* CueR at Various States during Transcription

**Figure 4:**
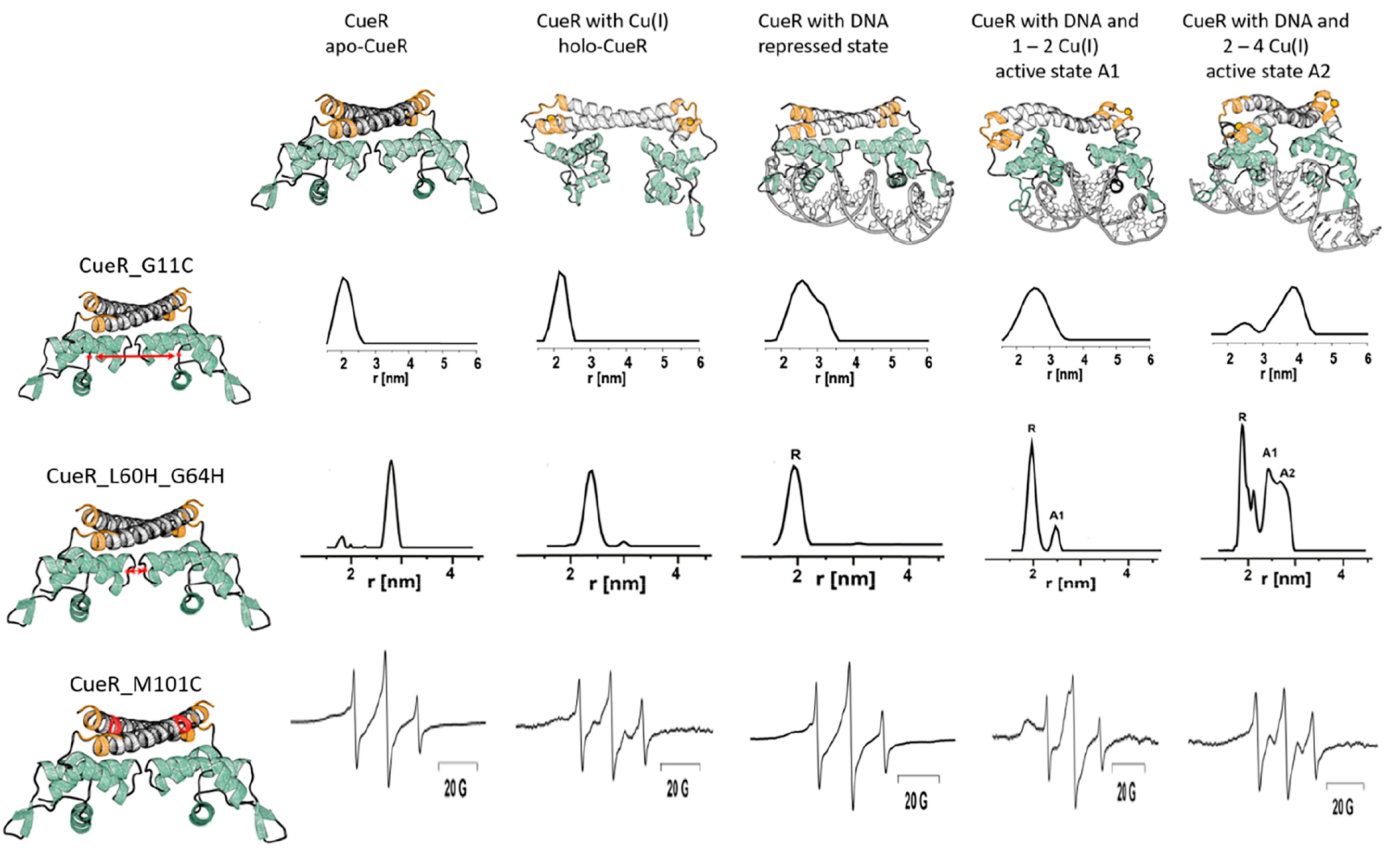
Changes in EPR distance distribution functions at the various states during transcription for CueR_G11C (adapted from ref (51) with permission, copyright 2017 Cell Press) and CueR_L60H_G64H (adapted from ref (50) with permission, copyright 2019 Wiley), and changes in CW-EPR spectra for the CueR_M101C mutant (adapted with permission from ref (13), copyright 2022 Wiley). The holo-CueR structures (active states A1and A2) at the top of the figure were developed using the distance distribution constraints and ENM models.

We exploited the benefits of DEER spectroscopy to target conformational changes that CueR assumes upon DNA and Cu(I) binding. Accordingly, we generated several mutant versions of CueR spin-labeled with MTSL so as to monitor distinct domains of the protein.^51^ The biochemical activity of the mutants was assessed with circular dichroism (CD), an electrophoresis mobility shift assay (EMSA), and pull-down experiments. DEER experiments were performed in the presence or absence of Cu(I) and DNA. For all mutants, changes between the apo-CueR state and the active CueR state (i.e., in the presence of Cu(I) and DNA) were detected, indicating that CueR undergoes conformational changes upon Cu(I) and DNA binding. However, a major change was detected for the CueR_G11C mutant, affected in the DNA-binding domain (Figure 4). DEER analysis of CueR_G11C revealed a change in the distance distribution function from 2.1 ± 0.3 nm in the apo-state to 2.2 ± 0.2 nm in the holo-form (bound to Cu(I)). In the repressed state (when CueR is bound to DNA), a broad distance distribution of 2.0–3.5 nm was found, and in this state, some of CueR molecules are bound to the DNA while others are unbound. Interestingly, in the presence of excess of Cu(I) (in the active state), a completely different conformational state of 3.8 ± 0.5 nm appeared. It is important to note that the latter conformational state is very broad, indicative of the fact that CueR can assume various conformational states in the presence of DNA and excess of Cu(I) ions. However, it was not possible to clearly distinguish between the various conformations.

### Increasing the Resolution of the DEER Data by Using Cu(II)–dHis Spin-Labeling

The MTSL spin-label approach is easy to use with respect to spin-labeling synthesis, labeling yield, and the absence of orientational affects that complicate data analysis. However, flexibility of the side chain affects resolution and the ability to distinguish between various conformational states. To increase the resolution between different active state conformations, we applied Cu(II)–dHis labeling.^50^ The L60H_G64H mutant affects the α4 helix of CueR, which connects the Cu(I)-binding domain with the DNA-binding domain. Figure 4 shows the DEER distance distribution detected at various states as a function of Cu(I) and DNA binding. With this spin-labeling methodology, a very narrow distance distribution function was noted, allowing the observation between the apo-repressed (named here R), and two active states (named here A1 and A2).

### Creating Structural Models Based on DEER Constraints

DEER constraints using MTSL and Cu(II)-dHis labeling allowed us to precisely predict the conformation of CueR in the apo-state and in the two active states (Figure 4). An elastic-network model (ENM) implemented in the multiscale modeling of macromolecular systems (MMM) software was applied, with the structure of copper-bound CueR (PDB 1Q05) as template for modeling. The models indicated that, in the active states, the two DNA-binding domains approach one other. In the A1 active state obtained at lower Cu(I) concentrations, the two DNA-binding domains were slightly closer than in the A2 active state, obtained at higher Cu(I) concentrations. In addition, we ran MD simulations for the apo- and holo-states based on the DEER constraints.^55^ These simulations suggested that the two DNA-binding domains can assume two kinds of dynamic states, namely, bending and twisting modes, which allows control of the DNA conformation.

### Beyond DEER: Additional EPR Methods That Can Shed Light on the Reaction Mechanism

Room temperature (RT) continuous wave (CW) EPR experiments coupled with nitroxide spin-labeling have been used for many years, beginning the 1960s, to study kinetics of biological systems. The obvious approach here is to introduce site-specific spin-labels into a macromolecule and deduce the mobility and dynamics of a domain derived from the EPR line shape. In our studies,^13^ we took RT CW EPR measurements to further explore the role of Cu(I) ions in the mechanism of action of CueR. We focused on two sites, namely, the Cu(I)-binding site, which was spin-labeled with MTSL on M101C, and the DNA-binding domain, which was spin-labeled on A16C. Distinct changes in the line shape were observed in the absence or presence of DNA as a function of Cu(I) concentration (Figure 4). Specifically, three regions were distinguished, in each region different mobility was detected. At a ratio of 0–1 Cu(I):CueR monomer, the CW-EPR profile suggested an increase in dynamics within the DNA-binding domain. At a ratio of 1–2 Cu(I):CueR, the CW-EPR spectra suggested limited dynamics in the Cu(I)-binding site, and an increase in dynamics of the DNA-binding domain. Addition of Cu(I) to the CueR solution resulted in an increase in dynamics of the entire protein. Integrating the CW-EPR data with the DEER data suggested that the A1 active conformation is less dynamic, especially the copper-binding domain, and, overall, is more compressed, based on the DEER data. This state probably allows initiation of the transcription process. Addition of more Cu(I) loosened some of the tight structure, which could potentially affect the transcription process.

### From the DNA Perspective

To be able to monitor conformational changes within the promoter itself during transcription, the Cu(II) DPA spin-labeling approach was used.^24^ DEER measurements were performed at different ratios of CueR:DNA in the absence or presence of Cu(I). DEER measurements on the DNA alone revealed a distribution around 4.2 nm. In the presence of 2:1 CueR:DNA, the distribution slightly changed to around 4.0 nm. However, in the presence of excess of CueR (at a 6:1 CueR:DNA ratio), this distance decreased to 3.6 nm. This either suggests low affinity between CueR and DNA or the fact that the presence of several CueR monomers can fold the DNA. In the presence of Cu(I), the affinity of CueR to the DNA increased, and even at a ratio of 2:1 CueR:DNA, distribution around 3.6 nm appeared, showing the key role of copper in activating transcription. However, it is important to note that, to better understand structural changes in the DNA, various spin-labeling positions should be considered.

### Final Remark: The Future of Structural Biology Using EPR Spectroscopy

EPR spectroscopy has proven to be a powerful tool for structural biology, specifically for resolving complex biological reaction mechanisms. We showed that distance EPR measurements can target minor conformational changes upon ligand or molecule binding that can reveal structural changes and dynamics which directly impact cellular function. Combining distance EPR results with changes in dynamics, as measured by RT CW EPR, along with other experimental and computational methods, will generate a comprehensive picture on the mechanism of action at the molecular level.

Although EPR measurements have drastically expanded the field of structural biology, many remaining aspects can be improved. These include the following:

- *Developing New Spin-Labeling Methodologies.* The development of new spin-labels, such as small probes able to penetrate narrow hydrophobic pores in the protein and spin-labels that can label amino acids such as lysine residues or unnatural amino acids, is needed. The latter will be less sensitive to reducing environments. Moreover, new spin-labeling approaches that are simple to synthesize by nonspecialized organic laboratories should also be developed.
- *In Cell EPR Problems and Mitigations.* In the past decade, *in cell* EPR methodologies have been successfully developed. Studying proteins within the cellular environment can reveal many biological mechanisms that have not yet been resolved. Because of the challenges associated with spin-labeling proteins within cells, *in cell* EPR measurements are performed on recombinant spin-labeled proteins, which are subsequently injected into the cellular environment. This procedure limits both the size of the protein of interest, as well as the cellular system, which is mostly applicable to eukaryotic systems, and for only one membrane. Therefore, new *in cell* spin-labeling methods are urgently needed to overcome the cumbersome steps of introducing exogenous labeled proteins.
- *EPR Sensitivity.* Since EPR measurements cannot be applied to single proteins but require overexpressed protein levels, the development of new EPR methodologies compatible with nanomolar protein concentrations offers tremendous potential and will open many new avenues in the field of structural biology.
